# Multigene PCR using both cfDNA and cfRNA in the supernatant of pleural effusion achieves accurate and rapid detection of mutations and fusions of driver genes in patients with advanced NSCLC

**DOI:** 10.1002/cam4.3769

**Published:** 2021-03-03

**Authors:** Xuejing Chen, Kun Li, Zichen Liu, Fei Gai, Guanshan Zhu, Shun Lu, Nanying Che

**Affiliations:** ^1^ Department of Pathology Beijing Chest Hospital, Capital Medical University, Beijing Tuberculosis And Thoracic Tumor Research Institute Beijing China; ^2^ Medical Department Amoy Diagnostics Co., Ltd. Xiamen China; ^3^ Department of Oncology Shanghai Chest Hospital Shanghai Jiaotong University Shanghai China

**Keywords:** *ALK* fusion, cfRNA, NSCLC, PCR, pleural effusion, supernatant

## Abstract

**Background:**

Pleural effusion from patients with advanced non‐small cell lung cancer (NSCLC) has been proved valuable for molecular analysis, especially when the tissue sample not available. However, simultaneous detection of multiple driver gene alterations especially the fusions is still challenging.

**Methods:**

In this study, 77 patients with advanced NSCLC and pleural effusion were enrolled, 49 of whom had matched tumor tissues. Supernatants, cell sediments, and cell blocks were prepared from pleural effusion samples for detection of driver alterations by a PCR‐based 9‐gene mutation detection kit.

**Results:**

Mutations in *EGFR, KRAS*, and *HER2* were detected in DNA and cfDNA, fusions in *ALK* was detected in RNA and cfRNA. Compared with matched tumor tissue, the supernatant showed the highest overall sensitivity (81.3%), with 81.5% for SNV/Indels by cfDNA and 80% for fusions by cfRNA, followed by cell blocks (71.0%) and the cell sediments (66.7%). Within the group of treatment‐naïve patients or malignant cells observed in the cell sediments, supernatant showed higher overall sensitivity (89.5% and 92.3%) with both 100% for fusions.

**Conclusions:**

CfDNA and cfRNA derived from pleural effusion supernatant have been successfully tested with a PCR‐based multigene detection kit. Pleural effusion supernatant seems a preferred material for detection of multigene alterations to guide treatment decision of advanced NSCLC.

## Graphical abstract

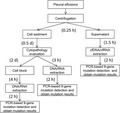

## 1 INTRODUCTION

Targeted therapies against driver gene alterations in patients with non‐small cell lung cancer (NSCLC) can significantly improve patient survival over conventional chemotherapy,^1^, ^2^, ^3^, ^4^, ^5^, ^6^ making molecular testing more important in standard clinical care. Today, therapies have been available for multiple actionable targets in NSCLC so that simultaneous detection of multigene alterations including both mutations and fusions has been routine clinical need. With the advancement of detection technology, detection of mutations based on tumor genomic DNA and detection of fusions based on tumor DNA or RNA can be achieved in a one‐shot test.^7^, ^8^ While tumor tissue sample for molecular profiling is the “gold standard” in clinical practice, it is not always available due to various reasons especially for patients with advanced clinical stage of the disease.^9^ Liquid biopsy samples including plasma, pleural effusions, cerebrospinal fluids, urine, and sputum have become promising alternative sample types for molecular test.^10^, ^11^, ^12^, ^13^ Peripheral blood cfDNA is regarded as the main type of liquid biopsy sample which has been used as complementary or surrogate sample for detecting driver gene alterations in NSCLC. Blood‐based liquid biopsy has the advantage in overcoming inter/intra tumor heterogeneity for advanced/metastatic NSCLC, while it also has a main shortcoming of low sensitivity in detecting driver gene mutations, especially for detecting fusions due to limited amount of qualified cfRNA.^14^, ^15^, ^16^

In clinic, a significant portion of patients with advanced NSCLC developed pleural effusions, especially when disease relapsed from prior treatments.^17^ Pleural effusions contain both floating malignant cells and cell free nucleic acid, which can be collected for diagnosis purposes.^18^ Multiple studies have reported that cell blocks derived from pleural effusions can be used to detect driver gene alterations as tumor tissue equivalent samples.^12^, ^19^ In addition, studies reported that the sensitivity of detecting *EGFR* mutations in the supernatant of pleural effusion were relatively independent from detectable malignant cells and blood cell contamination in pleural effusions.^20^, ^21^, ^22^, ^23^ However, supernatant of pleural effusions for multigene alterations testing with comparison to cell sediments, cell blocks, especially involving fusion detection of using cfRNA has not been fully investigated.

## 2 METHODS

### 2.1 Patients and samples

**FIGURE 1:**
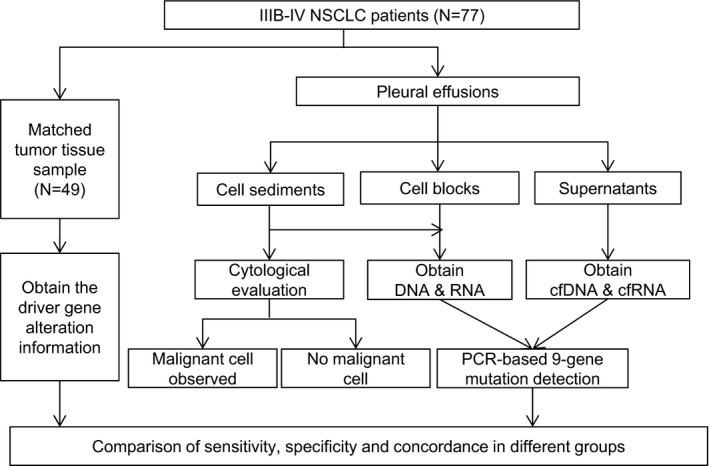
Flowchart of the analysis for driver gene alterations. cfDNA, cell free DNA; cfRNA, cell free RNA; NSCLC, non‐small cell lung cancer

The pleural effusions from 77 patients with advanced NSCLC were collected between September 2019 and May 2020 at the Beijing Chest Hospital, Beijing, China, among which matched tumor tissue samples were available from 49 patients. The information of clinicopathological characteristics of these patients was collected. Each pleural effusion specimen was centrifuged at 3000 g for 10 min and further processed into three types of components: supernatant, cell sediment, and cell block (formalin fixed paraffin embedded sample derived from half of the cell sediment). The supernatants and cell sediments were stored at −80°C and cell blocks were stored at room temperature until use. All the cell sediment specimens underwent cytology evaluation and classified as malignant cells observed or no malignant cell observed. The study was approved by the Ethical and Institutional Review Boards for Human Investigation of the Beijing Chest Hospital (Number: BJXK‐2020–08). The study design and procedure are presented in Figure 1.

### 2.2 DNA/RNA extraction

Genomic DNA and total RNA were extracted from cell sediment and cell block samples using FFPE DNA/RNA Extraction Kit (Amoy Diagnostics) according to the manufacturer's instructions. The cfDNA and cfRNA were extracted from supernatant samples simultaneously using QIAamp Circulating Nucleic Acid Kit (Qiagen) following manufacturer's instructions (Figure S1).

### 2.3 Molecular analyses

DNA and RNA were required for next molecular analyses for every sample. The concentration of DNA should be greater than 2 ng/μl, the concentration of RNA should be between 10 and 500 ng/μl. And the OD260/OD280 of DNA and RNA should be between 1.8 and 2.1. The molecular analysis of all samples was carried out by using AmoyDx^®^ Multi‐Gene Mutations Detection Kit (Amoy Diagnostics) according to the manufacturer's instructions. This kit contains DNA‐based mutation detection real‐time PCR assays and RNA‐based fusion detection real‐time PCR assays, which can detect 118 hotspot mutations/fusions in genes of *EGFR, KRAS, BRAF, NRAS, HER2, PIK3CA, ALK, ROS1, and RET* in a single test^8^ (Supplementary Method and Table S1).

### 2.4 Statistics analysis

Analyses were performed using SPSS (version 19.0). McNemar's test was used to assess the difference of detection rate between pleural effusion samples and matched tumor samples, *χ* ^2^ or Fisher's exact tests was used to assess the detection rate between different sample types. *p* < 0.05 was considered statistically significant (two‐sided). Graphic analysis was performed using GraphPad Prism 8.0 software (GraphPad Software, Inc.).

## 3 RESULTS

### 3.1 Patient characteristics

A total of 77 patients with advanced NSCLC who provided pleural effusion specimens were enrolled in this study (Table 1). The median age of these patients was 63.1 years old. Relatively, more patients were male (57.1%), with adenocarcinoma (88.3%), and treatment naive (66.2%), and with distant metastases (50.6%) (Table 1).

**TABLE 1:** Clinicopathological characteristics of 77 enrolled patients with advanced NSCLC

Characteristics (*N* = 77)	
Age	No. of patients (%)
Median (range)	63.1 ± 12.1
Sex	
Male	44 (57.1%)
Female	33 (42.9%)
Pathological evaluation of paired tumor samples	
Adenocarcinoma	68 (88.3%)
Squamous cell carcinoma	5 (6.5%)
Other non‐small cell lung cancer	4 (5.2%)
Patients’ treatment status	
Treatment naive	51 (66.2%)
Relapsed from prior treatment	22 (28.6%)
Unknown	4 (5.2%)
Metastases status	
Without metastases	9 (11.7%)
Local	24 (31.2%)
Distant	39 (50.6%)
Unknown	5 (6.5%)

### 3.2 Detection of driver gene alterations in different components of pleural effusion specimens from both treatment‐naïve patients and treatment relapsed patients

Driver gene alterations in *EGFR, KRAS, HER2*, and *ALK* were detected from pleural effusion specimens (for the details of alterations detected, see Table S2). Using driver gene alteration status in tumor tissue as reference, the supernatant, cell sediment, and cell block all showed specificity of 100%. The supernatant showed the highest overall sensitivity (81.3%, 95% CI 63.0%–92.1%), followed by cell block (71.0%, 95% CI 51.8%–85.1%), and the cell sediment (66.7%, 95% CI 48.1%–81.4%), although there was no statistically significant difference among the three groups (*p* = 0.273). For SNV/Indel mutations, supernatant cfDNAs demonstrated the highest sensitivity of 81.5% without significant difference from the tumor tissue samples, but the cell sediment DNAs and cell block DNAs exhibited similar sensitivities (68% vs. 66.7%) with significant difference from the tumor samples. For the detection of *ALK* fusions, the supernatant showed a sensitivity of 80% (95% CI 29.9%–98.9%) which was similar to that of cell block (83.3%) but superior to cell sediment (66.7%) (Table 2). *EGFR* T790M mutation was detected in supernatants from four patients with acquired resistance to 1st line EGFR‐TKI, among which only 1 cell block and 1 cell sediment were detected to be T790M positive (Table S3).

**TABLE 2:** Concordance of driver gene alteration status between tumor tissue and supernatants, cell sediments, or cell blocks

	Cell block (*N* = 45)		Cell sediment (*N* = 48)		Supernatant (*N* = 47)	
	Sensitivity	Specificity	Sensitivity	Specificity	Sensitivity	Specificity
SNV/InDels	68% (46.4%, 84.3%)	100% (80.0%, 100%)	66.7% (46.0%, 82.8%)	100% (80.8%, 100%)	81.5% (61.3%, 93.0%)	100% (80.0%, 100%)
Fusions	83.3% (36.5%, 99.1%)	100% (88.8%, 100%)	66.7% (24.1%, 94.0%)	100% (89.6%, 100%)	80% (29.9%, 98.9%)	100% (89.6%, 100%)
Overall	71.0% (51.8%, 85.1%)	100% (73.2%, 100%)	66.7% (48.1%, 81.4%)	100% (74.7%, 100%)	81.3% (63.0%, 92.1%)	100% (74.7%, 100%)

The supernatant samples achieved higher overall sensitivity of 89.5% in treatment naïve patients for the detection of mutations and fusions, compared with an overall sensitivity of 69.2% in patients relapsed from prior treatment, although there was no statistically significant difference between the two groups (*p* = 0.194). Cell block and cell sediment all showed similar overall sensitivities in treatment naïve patients and patients relapsed from prior treatment (70.6% vs. 71.4%, 64.3% vs. 69.2%). In treatment naïve patients, the sensitivities of SNV/Indels detection in cell block, sediment, and supernatant were 64.3%, 68.4%, and 88.2%, respectively, and the sensitivities of fusion detection in the three groups were all 100%. In patients relapsed from prior treatment, the sensitivities of SNV/Indels detection in cell block, sediment, and supernatant were 72.7%, 72.7%, and 70%, respectively, and the sensitivities of fusion detection in the three groups were all 66.7%, 33.3%, and 66.7%, respectively (Table 3).

**TABLE 3:** Effects of treatment status on the detection sensitivities of gene alterations in different types of pleural effusion samples

		Cell block (*N* = 45)	Cell sediment (*N* = 48)	Supernatant (*N* = 47)
SNV/InDels	Treatment naive	64.3% (35.6%, 86.0%)	68.4% (43.5%, 86.4%)	88.2% (62.3%, 97.9%)
	Relapsed from prior treatment	72.7% (39.3%, 92.7%)	72.7% (39.3%, 92.7%)	70% (35.4%, 91.9%)
Fusions	Treatment naive	100% (31.0%, 100%)	100% (31.0%, 100%)	100% (19.8%, 100%)
	Relapsed from prior treatment	66.7% (12.5%, 98.2%)	33.3% (1.8%, 87.5%)	66.7% (12.5%, 98.2%)
Overall	Treatment naive	70.6% (44.0%, 88.6%)	64.3% (35.6%, 86.0%)	89.5% (65.5%, 98.2%)
	Relapsed from prior treatment	71.4% (42.0%, 90.4%)	69.2% (38.9%, 89.6%)	69.2% (38.9%, 89.6%)

The false‐negative results of fusion detection were all in supernatants, cell sediments, and cell blocks from patients relapsed from prior TKI‐treatment. The false‐negative results of SNV/Indels detection in supernatant cfDNA were concentrated within patients relapsed from prior TKI treatment (3/5), while the false‐negative result of SNV/Indels detection in cell blocks and cell sediments were concentrated within treatment naïve patients (5/8, 6/9) (Table 3).

### 3.3 Effects of cytological evaluation results on the sensitivities of testing different types of pleural effusion specimens

In the samples with observed malignant cells, the sensitivities of SNV/Indels detection in cell block genomic DNA, sediment genomic DNA, and supernatant cfDNA were 85.0%, 85.7%, and 91.0%, respectively, and the sensitivities of fusion detection by testing RNAs from cell block and sediment and cfRNA from supernatant were all 100%. The overall sensitivities of testing cell block, sediment and supernatant for all alterations were 88%, 88%, and 92.3% in samples with observed malignant cells. In the samples without malignant cell, 40% sensitivity was achieved from testing supernatant for SNV/Indels detection but no positive result was achieved for all others (Table 4).

**TABLE 4:** Effects of cytological evaluation results on the detection sensitivities of gene alterations in supernatants, cell sediments, and cell blocks (tumor tissue as reference)

		Cell block (*N* = 45)	Cell sediment (*N* = 48)	Supernatant (*N* = 47)
SNV/InDels	Malignant cells observed	85% (61.1%, 96.0%)	85.7% (62.6%, 96.2%)	91.0% (69.4%, 98.4%)
	No malignant cell	0% (0%, 53.7%)	0% (0%, 48.3%)	40.0% (7.3%, 83.0%)
Fusions	Malignant cells observed	100% (46.3%, 100%)	100% (39.6%, 100%)	100% (39.6%, 100%)
	No malignant cell	0% (0%, 94.5%)	0% (0%, 80.2%)	0% (0%, 94.5%)
Overall	Malignant cells observed	88% (67.7%, 96.8%)	88% (67.7%, 96.8%)	92.3% (73.4%, 98.7%)
	No malignant cell	0% (0%, 48.3%)	0% (0%, 40.2%)	33.3% (6.0%, 75.9%)

### 3.4 The capability of molecular analysis for pleural effusion supernatant in patients with tumor tissue unavailable

Among 28 patients with no tissue samples available, 18 mutations (17 SNV/Indels, 1 fusion) could be detected using pleural effusion supernatant. And the detection rate of driver gene mutations in these patients was not significantly different from that of patients who provided the tumor tissue samples (Table 5).

**TABLE 5:** Comparison of alteration detection rate between using tumor tissue and using pleural effusion supernatant

	Driver gene alterations detection rate			
	Tumor tissue samples (*N* = 49)	Pleural effusion supernatants without paired tissue samples (*N* = 28)	All pleural effusion supernatants (*N* = 75)	*p*
SNV/InDels	28/49 (57.1%)	17/28 (60.7%)	39/75 (52%)	0.802
Fusions	6/49 (12.2%)	1/28 (3.6%)	5/75 (6.7%)	0.407

## 4 DISCUSSION

Tumor cells in patients with advanced lung cancer infiltrate pleural cavity via the hematogenous, direct, or lymphatic spread, resulting impaired lymphatic drainage and pleural effusion accumulation.^24^ Cytological examination of pleural effusion showed that 95% of malignant serous effusions represented metastatic disease, mainly composed of adenocarcinomas (70%–77%).^25^ In this study, 88.3% of the enrolled patients with lung adenocarcinoma and 81.8% of the patients with local or distant metastases, two characteristics that were similar to those reported in the literature. In addition, 36.4% (28/77) of the enrolled patients were diagnosed by pleural effusion cytology because tissue samples for diagnosis were not available. Therefore, pleural effusion samples are a valuable supplement for tumor tissue in pathological diagnosis.^26^

Molecular testing based on pleural effusion cytology specimens has been widely recommended in clinical guidelines of NSCLC and has become a routine method used in clinical practice.^24^, ^27^, ^28^ The mutation detection rates based on this kind of specimens are affected by tumor content.^21^, ^22^ Previous studies showed that pleural effusion supernatants contain more abundant tumor derived DNA (ctDNA) than pleural effusion sediments and plasma samples, and had higher detection rate of *EGFR* driver mutations especially for detection of T790M in *EGFR*‐TKI relapsed patients.^20^, ^22^ And several studies also showed that the somatic mutation detection sensitivity in supernatant cfDNA was independent of both tumor cellularity and pleural effusion appearance.^20^, ^22^ In our study, the SNV/Indels detection rate of supernatant was higher than that of cytology specimens (81.5% vs. 68.0% or 66.7%). A certain percentage of mutations were also detected in the supernatant of pleural effusion samples without malignant cells. However, in the group of patients with malignant cells observed in the pleural effusion, the SNV/Indels detection rate of cell blocks was increased from sensitivity of 68.0% to 85.0%, and the fusion detection rate was increased from 83.3% to 100%. Also, the SNV/Indels detection rate of cell sediment was increased from 65.7% to 85.7%, and the fusion detection rate was increased from 66.7% to 100%. This indicates the necessity of pathological assessment for molecular detection of pleural effusion cytology samples.

For the pleural effusion cfRNA detection, previous study showed that the pleural effusion cfRNA can successfully be extracted and achieving a 100% concordance rate of *ALK* fusion detection compared with cell blocks by PCR method.^29^ However, this study did not obtain matched tissue specimens as reference. Another study also showed the success rates of pleural effusion cell free total nucleic acid extraction and NGS sequencing have been verified to be higher than that of cell blocks by Xiang et al.^22^ In this study, we confirmed that fusions could be successfully detected in the cfRNA derived from MPE supernatant by using a PCR‐based method, achieving a concordance rate of 97.9% (46/47) with paired tissue samples. The results further strengthen the statement that the potential of pleural effusion specimens is superior to plasma specimens in detecting fusion variations.^30^, ^31^ In our study, more false‐negative mutations were detected in the supernatant of pleural effusion in patients who had previously received targeted therapy and progressed, including one patient with false‐negative in detection of *ALK* fusion. Although more *EGFR* T790M resistance mutations could be detected in the supernatant of pleural effusion specimens with malignant cells observed, suggesting that analysis may be necessary in combination with cytological evaluation for driver gene testing in patients relapsed from prior TKI treatment.

**FIGURE 2:**
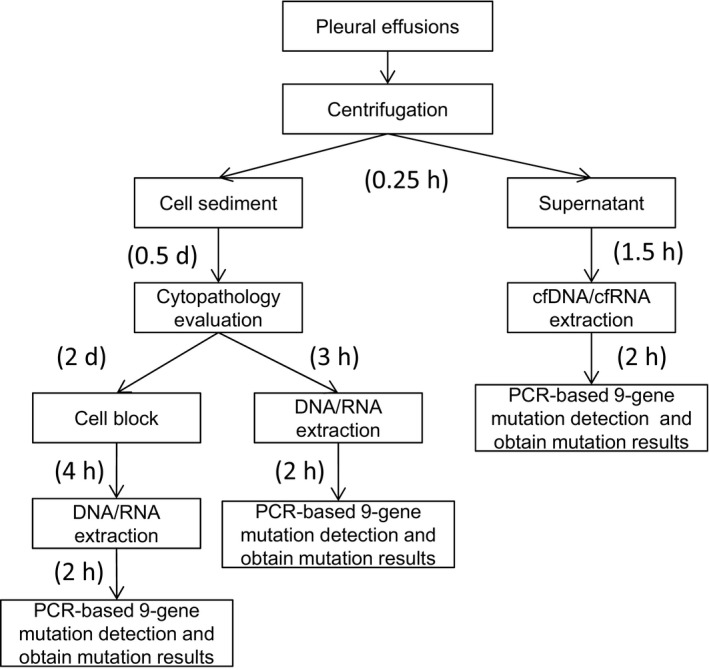
The workflow and TAT of different types of pleural effusion sample for driver gene alterations. cfDNA, cell free DNA; cfRNA, cell free RNA; d, days; h, hours; TAT, turnaround time

Use of cytology centrifuged supernatants of endobronchial ultrasound‐guided fine‐needle aspiration (EBUS‐guided FNA) could.improve cost and turnaround time for targeted next generation sequencing reported by Gokozan et al.^32^ In our study, the traditional workflow using cell blocks for molecular detection went through at least five steps, the TAT was at least 3 days. If the cell sediment was directly used for molecular detection, the TAT was at least one day. The workflow using supernatants for molecular detection was through three steps before got mutation results, the TAT was only 4 hours, which made obtain mutation results within one day possible in clinical practice. Compared with the direct molecular detection of cell sediments, the decreased TAT by the supernatant detection was mainly due to the omission of cytological evaluation. And compared with the cell block, the reduction in time of supernatant detection came from the omission of cytological evaluation and formalin fixing and paraffin embedding. So supernatant would be the preferred pleural effusion type for molecular detection, when consider the turnaround time and experiment steps (Figure 2).

There are several limitations in this study. First, the sample size is from a single center and not large enough, especially for the fusion variation positive samples. Further investigation in multiple centers with larger sample size is under planning to validate the results of this work. Second, in this study, we did not compare with matched blood specimens, and follow‐up studies will be conducted to explore the differences in fusion variations between pleural effusion specimens and matched plasma specimens. Third, cfRNA profile in MPE supernatant needs to be further investigated.

In conclusion, our study indicated that supernatant of pleural effusion specimens can achieve cfDNA‐based SNV/Indels and cfRNA‐based fusion variations detection. Compared with cytology specimens, the supernatant has the highest overall detection sensitivity, and the TAT of molecular testing is the shortest, the mutation results can be obtained on the day the pleural effusion is obtained. The entire detection process of pleural effusion supernatant was easy to operate and with short TAT, which was more suitable for clinical application.

## 5 ETHICS STATEMENT

The study was approved by the Ethical and Institutional Review Boards for Human Investigation of the Beijing Chest Hospital (Number: BJXK‐2020–08).

## CONFLICT OF INTEREST

Guanshan Zhu is the stock holder of Amoy Diagnostics.

## Supporting information

Supplementary Material

## Data Availability

Some or all data generated or used during the study are available from the corresponding author by request.
