# Supplementary material for: Multigene PCR using both cfDNA and cfRNA in the supernatant of pleural effusion achieves accurate and rapid detection of mutations and fusions of driver genes in patients with advanced NSCLC
Source: Cancer Med. 2021 Mar 3;10(7):2286–92. doi: 10.1002/cam4.3769 (PMC7982639; doi:10.1002/cam4.3769)
Supplement: Supplementary file 1 — Supplementary Material [file CAM4-10-2286-s001.docx]

**Methods**

**Molecular Analyses**

Sample quality control

After the separation of DNA and RNA, the concentration of DNA and RNA was measured using a micro-ultraviolet spectrophotometer. The concentration of RNA should be between 10-500 ng/ɥL, the concentration of DNA should be greater than 2 ng/ɥL. And the OD_260_/OD_280_ of DNA and RNA should be between 1.8-2.1. The sample was tested immediately after the measurement completed.

Technological Principles

The assay contains DNA-based mutation detection and mRNA-based fusion detection.

The RNA gene fusion detection includes two processes: 1) Reverse Transcription: extracted RNA from FFPE or fresh tumor tissue is employed in this step, reverse transcription of target RNA enables complementary DNA (cDNA) synthesis with the action of reverse transcriptase and specific primers. 2) PCR Amplification: the specific primers are designed for amplification of cDNA, and ALK, ROS1, RET, MET, NTRK1, NTRK2 and NTRK3 variant amplicon is detected by fluorescent probes.

The DNA gene mutation detection system uses ARMS technology, which comprises specific primers and fluorescent probes to detect gene mutations. During the amplification, the target mutant DNA is matched with the bases at 3’ end of the primer, and amplified efficiently, then the mutant amplicon is detected by fluorescent-labeled probes. While the wild-type DNA cannot be matched with specific primers, there is no amplification occurs. By simultaneously detecting mutations at multiple sites in a single tube, the detection of more than 100 hotspot variants in *EGFR/ ALK/ ROS1/ KRAS/ BRAF/ HER2/ RET/ PIK3CA/ NRAS* genes at one time.

**Table S1. Details of the molecular analyses used in this study**

| **Tube** | **Target** | **Fusion Type** |
| --- | --- | --- |
| Tube 1  FAM | ALK | EML4 exon13; ALK exon 20 |
|  |  | EML4 exon 6 ins33; ALK exon 20 |
|  |  | EML4 exon 20; ALK exon 20 |
|  |  | EML4 exon 18; ALK exon 20 |
|  |  | EML4 exon 2; ALK exon 20 |
|  |  | EML4 exon 17; ins68 ALK exon 20 |
|  |  | EML4 exon 2; ins117 ALK exon 20 |
|  |  | EML4 exon 13; ins69 ALK exon 20 |
|  |  | EML4 exon 6; ALK exon 20 |
|  |  | EML4 exon 6; ALK exon 19 |
|  |  | EML4 exon 6; ins18 ALK exon 20 |
|  |  | EML4 exon 20; ins18 ALK exon 20 |
|  |  | EML4 exon 17 del58; ins 39 ALK exon 20 |
|  |  | EML4 exon 17 ins 65; ALK exon 20 |
|  |  | EML4 exon 17; ins 30 ALK exon 20 |
|  |  | EML4 exon 17 ins61; ins 34 ALK exon 20 |
|  |  | EML4 exon 3; ins53 ALK exon 20 |
| Tube 2  FAM | ROS1 | SLC34A2 exon4; ROS1 exon 32 |
|  |  | SLC34A2 exon14 del; ROS1 exon 32 |
|  |  | CD74 exon6; ROS1 exon 32 |
|  |  | SDC4 exon2; ROS1 exon 32 |
|  |  | SDC4 exon4; ROS1 exon 32 |
|  |  | SLC34A2 exon4; ROS1 exon 34 |
|  |  | SLC34A2 exon14 del; ROS1 exon 34 |
|  |  | CD74 exon6; ROS1 exon 34 |
|  |  | SDC4 exon4; ROS1 exon 34 |
|  |  | EZR exon10; ROS1 exon 34 |
| Tube 3  FAM | ROS1 | TPM3 exon 8; ROS1 exon 35 |
|  |  | LRIG3 exon 16; ROS1 exon 35 |
|  |  | GOPC exon 8; ROS1 exon 35 |
| Tube 4  FAM | RET | CCDC6 exon 1; RET exon 12 |
|  |  | NCOA4 exon6; RET exon 12 |
|  |  | KIF5B exon 15; RET exon 12 |
|  |  | KIF5B exon 16; RET exon 12 |
|  |  | KIF5B exon 23; RET exon 12 |
|  |  | KIF5B exon 22; RET exon 12 |

|  | **Exon** | **Mutation name** | **Base change** |
| --- | --- | --- | --- |
| Tube 5  FAM | EGFR exon 19 | E746_A750del (1) | 2235_2249del15 |
|  |  | E746_A750del (2) | 2236_2250del15 |
|  |  | L747_P753>S | 2240_2257del18 |
|  |  | E746_T751>I | 2235_2252>AAT(complex) |
|  |  | E746_T751del | 2236_2253del18 |
|  |  | E746_T751>A | 2237_2251del15 |
|  |  | E746_S752>A | 2237_2254del18 |
|  |  | E746_S752>V | 2237_2255>T(complex) |
|  |  | E746_S752>D | 2238_2255del18 |
|  |  | L747_A750>P | 2238_2248>GC(complex) |
|  |  | L747_T751>Q | 2238_2252>GCA(complex) |
|  |  | L747_E749del | 2239_2247delTTAAGAGAA |
|  |  | L747_T751del | 2239_2253del15 |
|  |  | L747_S752del | 2239_2256del18 |
|  |  | L747_A750>P | 2239_2248TTAAGAGAAG>C(complex) |
|  |  | L747_P753>Q | 2239_2258>CA(complex) |
|  |  | L747_T751>S | 2240_2251del12 |
|  |  | L747_T751del | 2240_2254del15 |
|  |  | L747_T751>P | 2239_2251>C(complex) |
|  |  | L747_T751del | 2238_2252del15 |
|  |  | L747_S752>Q | 2239_2256>CAA(Complex) |
|  |  | L747_A750>P | 2239_2250>CCC(Complex) |
|  |  | L747_K754>QL | 2239_2261>CAATT(Complex) |
|  |  | E746_K754>EQHL | 2238_2261>GCAACATCT(Complex) |
|  |  | L747_S752>Q | 2238_2256>GCAA (Complex) |
| Tube 5  VIC | EGFR exon 20 | S768I | 2303G>T |
| Tube 6  FAM | EGFR exon 21 | L858R | 2573T>G |
| Tube 6  VIC | EGFR exon 18 | G719A | 2156G>C |
|  |  | G719S | 2156G>A |
|  |  | G719C | 2156G>T |
| Tube 7  FAM | EGFR exon 20 | T790M | 2369C>T |
| Tube 7  VIC | EGFR exon 21 | L861Q | 2582T>A |
| Tube 8  FAM | KRAS exon 2 | G12D | 35G>A |
|  |  | G12S | 34G>A |
| Tube 8  VIC | BRAF exon 15 | V600E | 1799T>A |
|  |  | V600K | 1798_1799GT>AA(complex) |
|  |  | V600E2 | 1799_1800TG>AA (complex) |
|  |  | V600R | 1798_1799GT>AG(complex) |
|  |  | V600D1 | 1799_1800TG>AC(complex) |
|  |  | V600D2 | 1799_1800TG>AT(complex) |
| Tube 9  FAM | KRAS exon 2 | G12A | 35G>C |
|  |  | G12V | 35G>T |
|  |  | G12R | 34C>T |
|  |  | G12C | 34G>T |
|  |  | G13C | 37G>T |
| Tube 9  VIC | HER2 exon 20 | A775_G776insYVMA | 2325_2326 ins12 (TACGTGATGGCT) |
|  |  | A775_G776insYVMA | 2324_2325 ins12 (ATACGTGATGGC) |
|  |  | M774_A775insAYVM | 2322_2323ins12 (GCATACGTGATG) |
| Tube10  FAM | NRAS exon 3 | Q61R | 182A>G |
|  |  | Q61K | 181C>A |
|  |  | Q61L | 182A>T |
|  |  | Q61H | 183A>C |
| Tube10  VIC | HER2 exon 20 | G776>VC | 2326_2327ins3 (TGT) |
|  |  | P780_Y781insGSP | 2339_2340 ins9 (TGGCTCCCC) |
|  |  | P780_Y781insGSP | 2339_2340ins9 (GGGCTCCCC) |
|  |  | P780_Y781insGSP | 2340_2341ins9 (GGCTCCCCA) |
|  |  | G776>VC | 2326_2327ins3 (TTT) |
|  |  | P780_Y781insGSP | 2339_2340ins9 (CGGCTCCCC) |
|  |  | G776>VC | 2326_2327insTAT |
|  |  | G776>VC | 2326_2327insTCT |
|  |  | G776>LC | 2326G>TTAT |
| Tube11  FAM | EGFR exon 20 | H773_V774insH | 2319_2320insCAC |
|  |  | D770_N771insG | 2310_2311insGGT |
|  |  | V769_D770insASV | 2307_2308insGCCAGCGTG |
|  |  | D770_N771insSVD | 2311_2312insGCGTGGACA |
|  |  | V769_D770insASV | 2309_2310AC>CCAGCGTGGAT |
|  |  | H773_V774insNPH | 2319_2320insAACCCCCAC |
|  |  | H773_V774insQ | 2319_2320insCAG |
|  |  | N771_P772insT | 2313_2314insACC |
|  |  | N771_P772insH | 2314_2315insACC |
|  |  | P772_H773insQ | 2318_2319insACA |
|  |  | H773_V774insY | 2319_2320insTAC |
|  |  | V769_D770insGSV | 2308_2309insGCAGCGTGG |
|  |  | D770_N771insG | 2310_2311insGGG |
|  |  | D770_N771insG | 2310_2311insGGC |
|  |  | P772_H773insDNP | 2307_2308insGACAACCCC |
| Tube11  VIC | PIK3CA exon 20 | H1047R | 3140A>G |
|  | PIK3CA exon 9 | E545K | 1633G>A |

**Results**

**Table S2 Detailed results of all samples tested for driver gene alterations**

|  | | EGFR sensitive mutations (n) | ALK fusions (n) | Other SNV/InDels (n) |
| --- | --- | --- | --- | --- |
| Tumor tissue samples (N=49) | | 19del (14)  L858R(7)  20ins (2) | EML4-ALK (6) | KRAS mutation (4)  HER2 20ins (1) |
| Pleural effusions  (N=77) | Cell blocks  (N=74) | 19del (9)  L858R(14)  20ins (3)  G719X(1) | EML4-ALK (6) | KRAS mutation (2)  BRAF mutation (3) |
|  | Cell sediment  (N=76) | 19del (10)  L858R(14)  20ins (2)  G719X(1) | EML4-ALK (5) | KRAS mutation (2)  HER2 20ins (1)  BRAF mutation (3) |
|  | Supernatants  (N=75) | 19del (14)  L858R(15)  20ins (3)  G719X(1) | EML4-ALK (5) | KRAS mutation (2)  HER2 20ins (1)  BRAF mutation (3) |

| Patients ID | Tissue | Pleural effusions | | |
| --- | --- | --- | --- | --- |
|  |  | Cell block | Cell sediment | Supernatant |
| 1 | NA | EGFR L858R | EGFR L858R | EGFR L858R |
| 2 | KRAS mutation | KRAS mutation | KRAS mutation | NA |
| 3 | EGFR 19-Del | NA | NA | EGFR 19-Del |
| 4 | EML4-ALK fusion | EML4-ALK fusion | EML4-ALK fusion | EML4-ALK fusion |
| 5 | EGFR 19-Del | EGFR 19-Del | EGFR 19-Del | EGFR 19-Del |
| 6 | NA | BRAF mutation | BRAF mutation | BRAF mutation |
| 7 | EGFR 19-Del | NA | EGFR 19-Del | EGFR 19-Del |
| 8 | EGFR 19-Del | Negative | Negative | EGFR 19-Del |
| 9 | NA | EGFR L858R | EGFR L858R | EGFR L858R |
| 10 | Negative | Negative | Negative | Negative |
| 11 | NA | EGFR L858R | EGFR L858R | EGFR L858R |
| 12 | NA | EGFR 20INS | EGFR 20INS | EGFR 20INS |
| 13 | KRAS mutation | KRAS mutation | KRAS mutation | KRAS mutation |
| 14 | NA | Negative | Negative | Negative |
| 15 | NA | EGFR G719X | EGFR G719X | EGFR G719X |
| 16 | NA | Negative | Negative | Negative |
| 17 | EGFR 19-Del | EGFR 19-Del | EGFR 19-Del/T790M | EGFR 19-Del/T790M |
| 18 | KRAS mutation | Negative | Negative | Negative |
| 19 | NA | EGFR L858R | EGFR L858R | EGFR L858R |
| 20 | Negative | Negative | Negative | Negative |
| 21 | EGFR 19-Del | EGFR 19-Del | EGFR 19-Del | EGFR 19-Del |
| 22 | Negative | Negative | Negative | Negative |
| 23 | NA | BRAF mutation | BRAF mutation | BRAF mutation |
| 24 | EGFR 19-Del/T790M | EGFR 19-Del/T790M | EGFR 19-Del/T790M | EGFR 19-Del/T790M |
| 25 | EGFR 19-Del | EGFR 19-Del | EGFR 19-Del | EGFR 19-Del |
| 26 | Negative | Negative | Negative | Negative |
| 27 | Negative | Negative | Negative | Negative |
| 28 | EGFR L858R | EGFR L858R | Negative | EGFR L858R |
| 29 | NA | EML4-ALK fusion | EML5-ALK fusion | EML6-ALK fusion |
| 30 | NA | Negative | EGFR L858R | EGFR L858R |
| 31 | EGFR 20-ins | Negative | Negative | Negative |
| 32 | Negative | Negative | Negative | Negative |
| 33 | EGFR 19-Del | EGFR 19-Del | EGFR 19-Del | EGFR 19-del/T790M |
| 34 | EGFR L858R | EGFR L858R | EGFR L858R | EGFR L858R |
| 35 | EGFR 20-ins | EGFR 20-ins | EGFR 20-ins | EGFR 20-ins |
| 36 | NA | Negative | Negative | EGFR L858R |
| 37 | NA | EGFR L858R | EGFR L858R | EGFR L858R |
| 38 | HER2 20-ins | Negative | HER-2 20INS | HER-2 20INS |
| 39 | Negative | Negative | Negative | Negative |
| 40 | EML4-ALK fusion | EML4-ALK fusion | Negative | Negative |
| 41 | EGFR 19-Del | NA | Negative | Negative |
| 42 | Negative | Negative | Negative | Negative |
| 43 | EML4-ALK fusion | EML4-ALK fusion | EML4-ALK fusion | NA |
| 44 | NA | Negative | Negative | Negative |
| 45 | NA | Negative | Negative | Negative |
| 46 | EGFR 19-Del | EGFR 19-Del | EGFR 19-Del | EGFR 19-Del |
| 47 | NA | Negative | Negative | Negative |
| 48 | NA | Negative | Negative | Negative |
| 49 | NA | EGFR L858R | EGFR L858R | Negative |
| 50 | NA | BRAF mutation | BRAF mutation | BRAF mutation |
| 51 | Negative | Negative | Negative | Negative |
| 52 | EGFR L858R | EGFR L858R | EGFR L858R | EGFR L858R |
| 53 | EGFR L858R | EGFR L858R | EGFR L858R | EGFR L858R |
| 54 | EGFR L858R | EGFR L858R | EGFR L858R | EGFR L858R |
| 55 | NA | EGFR L858R | EGFR L858R | EGFR L858R |
| 56 | EGFR L858R | EGFR L858R | EGFR L858R | EGFR L858R |
| 57 | Negative | Negative | Negative | Negative |
| 58 | Negative | Negative | Negative | Negative |
| 59 | NA | Negative | Negative | EGFR 19-Del |
| 60 | NA | Negative | Negative | Negative |
| 61 | NA | EGFR 19-Del | EGFR 19-Del | EGFR 19-Del |
| 62 | EGFR 19-Del | EGFR 19-Del | EGFR 19-Del | EGFR 19-del/T790M |
| 63 | NA | Negative | Negative | Negative |
| 64 | EML4-ALK fusion | EML4-ALK fusion | Negative | EML4-ALK fusion |
| 65 | Negative | Negative | Negative | Negative |
| 66 | NA | Negative | Negative | Negative |
| 67 | EGFR L858R | Negative | Negative | Negative |
| 68 | EGFR 19-Del | Negative | Negative | Negative |
| 69 | Negative | NA | Negative | Negative |
| 70 | KRAS mutation | Negative | Negative | KRAS mutation |
| 71 | EML4-ALK fusion | EML4-ALK fusion | EML4-ALK fusion | EML4-ALK fusion |
| 72 | Negative | Negative | Negative | Negative |
| 73 | NA | EGFR 20INS | EGFR 20INS | EGFR 20INS |
| 74 | NA | EGFR L858R | EGFR L858R | EGFR L858R |
| 76 | EGFR 19-Del | Negative | Negative | EGFR 19-Del |
| 77 | Negative | Negative | Negative | Negative |
| 78 | EML4-ALK fusion | Negative | EML4-ALK fusion | EML4-ALK fusion |

NA: not available; Negative: no detected mutation in the 9 genes

**Table S3 Details of four patients with EGFR T790M mutation**

| Sample ID | Patients’ treatment status | Cell block | cell sediment | Supernatants |  |
| --- | --- | --- | --- | --- | --- |
| 17 | 1^st^ EGFR TKI progress | 19-del | 19-del/T790M | 19-del/T790M |  |
| 24 |  | 19-del/T790M | 19-del | 19-del/T790M | Plasma cfDNA 19-del/T790M |
| 33 |  | 19-del | 19-del | 19-del/T790M |  |
| 62 |  | 19-del | 19-del | 19-del/T790M |  |

**Figure s1** Fragment size analysis by Qsep100 Automatic nucleic acid analysis system. Size distribution of cfDNA recovered from cases 1 (A), 2 (B), respectively. bp, base pairs; RFU, relative fluorescence units


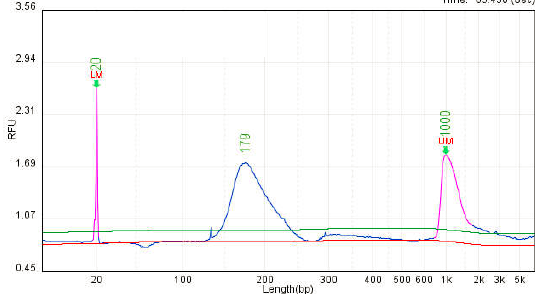


A


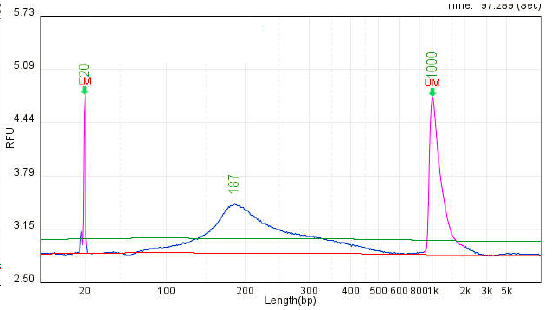


B
